# To Every Thing There Is a Season: Phenology and Photoperiodic Control of Seasonal Development in the Invasive Caucasian Population of the Brown Marmorated Stink Bug, *Halyomorpha halys* (Hemiptera: Heteroptera: Pentatomidae)

**DOI:** 10.3390/insects13070580

**Published:** 2022-06-25

**Authors:** Sergey Ya. Reznik, Natalia N. Karpun, Vilena Ye. Zakharchenko, Yelena I. Shoshina, Margarita Yu. Dolgovskaya, Aida Kh. Saulich, Dmitry L. Musolin

**Affiliations:** 1 Zoological Institute of the Russian Academy of Sciences, Universitetskaya Nab. 1, 199034 Saint Petersburg, Russia; reznik1952@mail.ru (S.Y.R.); bcongroup@gmail.com (M.Y.D.); 2 Federal Research Centre the Subtropical Scientific Centre of the Russian Academy of Sciences, Yana Fabritsiusa Street 2/28, 354002 Sochi, Russia; nkolem@mail.ru (N.N.K.); vilena.p2016@mail.ru (V.Y.Z.); haska6767@mail.ru (Y.I.S.); 3 Department of Forest Protection, Wood Science and Game Management, St. Petersburg State Forest Technical University, Institutskiy per. 5, 194021 Saint Petersburg, Russia; 4 Department of Entomology, St. Petersburg State University, Universitetskaya Nab. 7–9, 199034 Saint Petersburg, Russia; 325mik40@gmail.com

**Keywords:** brown marmorated stink bug, development, diapause, *Halyomorpha halys*, invasive pests, phenology, photoperiodic response, photoperiodism, reproduction, voltinism

## Abstract

**Simple Summary:**

The brown marmorated stink bug originates in East Asia and recently invaded Europe and North America. It is considered a serious pest because it damages 300+ species of wild and cultivated crops. Studies on the seasonal development of local populations of invasive insects are important for monitoring damage and predicting their dispersion. We investigated the seasonal development of this pest in Sochi (Krasnodar Territory, Russia) from 2018 to 2021 by regular field sampling. The results suggest that the brown marmorated stink bug normally produces two generations per year in the studied region: the main period when overwintered females lay eggs occurs from June to July; the second period of egg-laying (by females of the new generation) occurs in August. Reproductively active adult bugs were recorded from the end of May to the beginning of September. Such seasonal activity correlates with day length: when days became shorter than the experimentally determined critical value (15.0–15.5 h), the proportion of females with fully developed reproductive organs sharply dropped to zero. The timing of the beginning of the winter dormancy observed under the natural conditions agrees with the predictions based on the results of the earlier conducted laboratory experiments.

**Abstract:**

Studies on the phenology of local populations of invasive insects are necessary for monitoring and predicting their dispersion. We investigated the phenology of the brown marmorated stink bug, *Halyomorpha halys*, in the Sochi region (Krasnodar Territory, Russia) from 2018 to 2021 by regular field sampling and dissecting. The results of the sampling suggest that *H.* *halys* is at least partially bivoltine in the studied region: the main period of mass oviposition (by the overwintered females) occurs from June to July; the second, much shorter period of egg-laying (by females of the new, i.e., the first generation) occurs in August. Reproductively active individuals (i.e., females with developed ovaries and filled spermatheca and males with filled ectodermal sac) were recorded from the end of May to the beginning of September. Such a seasonal pattern correlated with day length: when the natural photoperiod decreased below the experimentally determined critical day length (15.0–15.5 h), the proportions of females with fully developed ovaries sharply dropped to zero. Both the rate of *H. halys* pre-adult development and the timing of the induction of winter adult diapause observed under natural conditions fully agreed with the earlier predictions that had been based on the results of laboratory experiments.

## 1. Introduction

All natural habitats are subjected to a certain extent to seasonal changes in environmental conditions. In accordance with these changes, insects realize their seasonal cycles, ensuring survival through the adverse periods as well as successful development and reproduction during favorable seasons. The patterns of natural yearly changes in environmental conditions vary markedly in different climates, and therefore, any seasonal insect cycle is fully adapted to the local climate only. Thus, geographically distant populations of widely distributed insect species can differ in voltinism, the timing of diapause induction, and many other ecophysiological traits [1,2,3,4,5]. Invasive pests, intentionally introduced as biocontrol agents, and other insects dispersed outside their initial native ranges need to rapidly adapt their developmental cycles to the new seasonal dynamics of the environment. The seasonal cycles of such populations can change noticeably within a comparatively short time [4,6,7]. Verified data on the actually realized seasonal pattern are necessary for monitoring population density, justifying the timing of control measures, and predicting the possible further dispersion of an invasive insect pest. Therefore, studies on the phenology of local populations of invasive insects are of high practical importance.

The present study focuses on the brown marmorated stink bug, *Halyomorpha halys* (Stål, 1855) (Hemiptera: Heteroptera: Pentatomidae). This East Asian invader is now considered one of the most serious alien pests in North America and Europe [8,9,10,11,12,13]. Recently *H. halys* was recorded on the northern coast of the Black Sea: in Abkhazia and the Krasnodar Territory of Russia [13,14,15,16]. The phenology and seasonal cycle of *H. halys* have been studied in various regions [4,17,18,19,20,21,22,23,24,25,26,27,28,29]. It is known that although the brown marmorated stink bug is potentially multivoltine, within its native range in China, Korea, and Japan, it produces only one or two generations per year. In such cases, overwintered females from bivoltine populations start to lay eggs in May, whereas adults of the first generation oviposit from June to July [30]. In North America, the invasive populations of this species are also bivoltine or at least partially bivoltine [19,29,31,32,33,34,35,36]. In Italy, *H. halys* produces two generations per year as well, and overwintered bugs lay eggs from May to August, and females of the first generation do so from July to September [37], whereas the Swiss population is univoltine, the oviposition period lasts from June to September [19]. The voltinism of the Caucasian invasive population has never been studied; however, sporadic earlier field observations suggested that in the Sochi region, *H. halys* can produce at least two generations per year [15].

The main aim of the present study was to support these estimations with the results of regular field sampling. In addition, we aimed to compare the observed phenology of the brown marmorated stink bug with the results of earlier laboratory experiments [16] and thereby evaluate the usefulness of laboratory data for modeling insect development under natural conditions, as this issue has been widely discussed in insect ecology and physiology [38,39,40,41,42].

## 2. Materials and Methods

**Figure 1:**
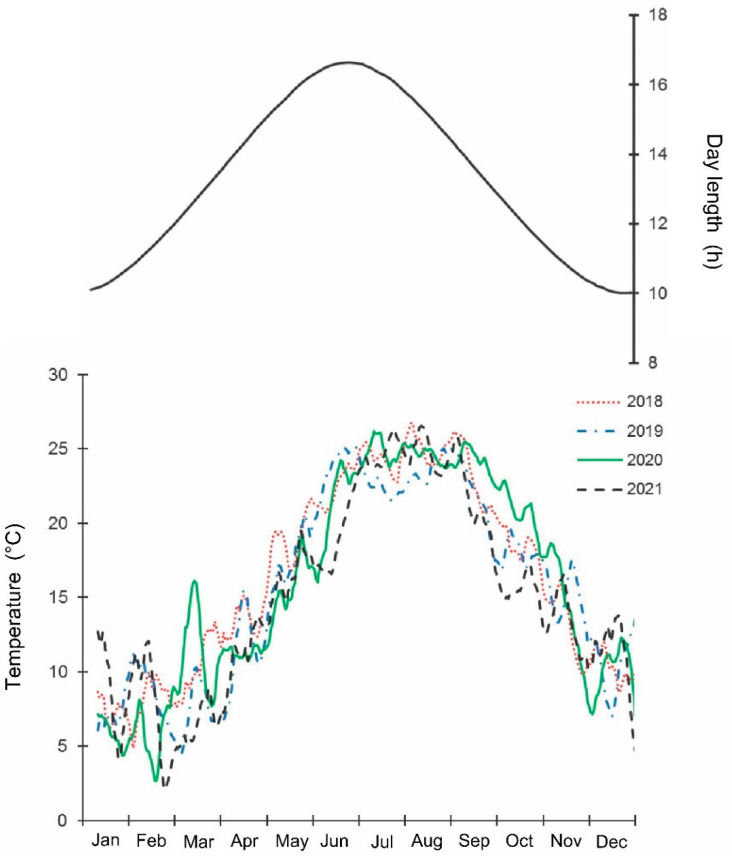
Seasonal changes in temperature and day length in the studied region of Sochi during 2018–2021. Upper graph, right vertical axis—day length including civil twilight [45] https://ru.365.wiki/world/russia/sochi/sun/calendar/ (accessed on 12 May 2022). Lower graph, left vertical axis—mean daily temperature; the lines are moving averages of 10 points (produced in Excel 2016) [46] http://www.pogodaiklimat.ru (accessed on 12 May 2022).

The samples were collected weekly by one trained person, by hand, from May 2018 until September 2021 along a 4 km long transect in the Khosta district, Sochi City, Krasnodar Territory of Russia (ca. 43.57° N; 39.75° E; 5–100 m a.s.l.). The collection usually took about 4 h. The collector observed as many individual trees and shrubs of different species as possible (mostly leaves (from both sides), flowers, and fruits; normally 3–5 full branches per plant); if a tree was taller than 2 m, then the lover part of the tree was examined. The transect crossed various habitats: parks, private gardens, fruit orchards, etc. All stages, from egg masses to adults, were collected. The studied region has a wet subtropical climate: the total annual precipitation is about 1500 mm; the average temperatures of January and August are +6.0 °C and +24.6 °C, correspondingly; the year average temperature is +14.5 °C; the no-frost period is about 300 days [43,44]. The mean, max, and min daily temperatures and day length in the studied region during 2018–2021 are shown in Figure 1 and Table S1, from which it is apparent that the general seasonal dynamics of temperature within this period were similar, but summer temperatures were somewhat higher in 2018 and lower in 2021 than in other years. Indeed, the mean temperatures for May–September in 2018, 2019, 2020, and 2021 were 23.2, 22.5, 22.9, and 21.9 °C, correspondingly [44]. The raw data for all figures are presented in Tables S1–S8.

During the warm season (May–September), *H. halys* egg batches, nymphs, and adults were collected; during the rest of the year (October–April), only adults (females and males) were collected. Females and males collected between June 2018 and June 2021 were taken to the laboratory, killed in a freezer, and dissected. At the time of dissection, the state of the reproductive organs and fat body of females and males were evaluated based on the criteria commonly used and proved reliable data for *H. halys* and other pentatomids [27,35,47,48,49,50,51,52,53,54,55]. To describe the seasonal dynamics of the state of reproductive organs and fat body with acceptable preciseness, we used three categories (or degrees) in each of the following cases:

− The development of the ovaries of females: (1) fully developed (reproductive state; mature eggs or vitellogenic oocytes in ovarioles), (2) partially developed (transitional state; initial stages of development of ovarioles); and (3) not developed (nonreproductive state; no visible signs of development of ovarioles);
− The fullness of the spermatheca of females: (1) full (a spermatheca is large, full, and has a spherical shape); (2) partly filled (intermediate state; a spermatheca is somewhat enlarged but filled only partly); and (3) empty (a spermatheca is small and shrunk);
− The development of the ectodermal sacs of accessory glands of males: (1) full (reproductive state; ectodermal sacs are fully filled with secretory fluids), (2) partly filled (ectodermal sacs are only partly filled with secretory fluids); and (3) empty (nonreproductive state; ectodermal sacs are not filled with secretory fluids and shrunk);
− The development of fat bodies of females and males: (1) fully developed (massive, abundant, or dense; composed of well-formed globules and interconnected stringy lobes), (2) moderately developed (intermediate, develop to a limited extent; globules have different size); and (3) poorly developed (lean, practically inconspicuous, or depleted).

In total, 40 egg batches, 1488 nymphs, and 2969 adults were collected (1180 females and 1154 males were then dissected). For statistical analysis of the proportions, Spearman’s correlation coefficient and the chi-square test were used. The data were analyzed with SYSTAT 10.2 (Systat Software Inc., Richmond, VA, USA).

## 3. Results

**Figure 2:**
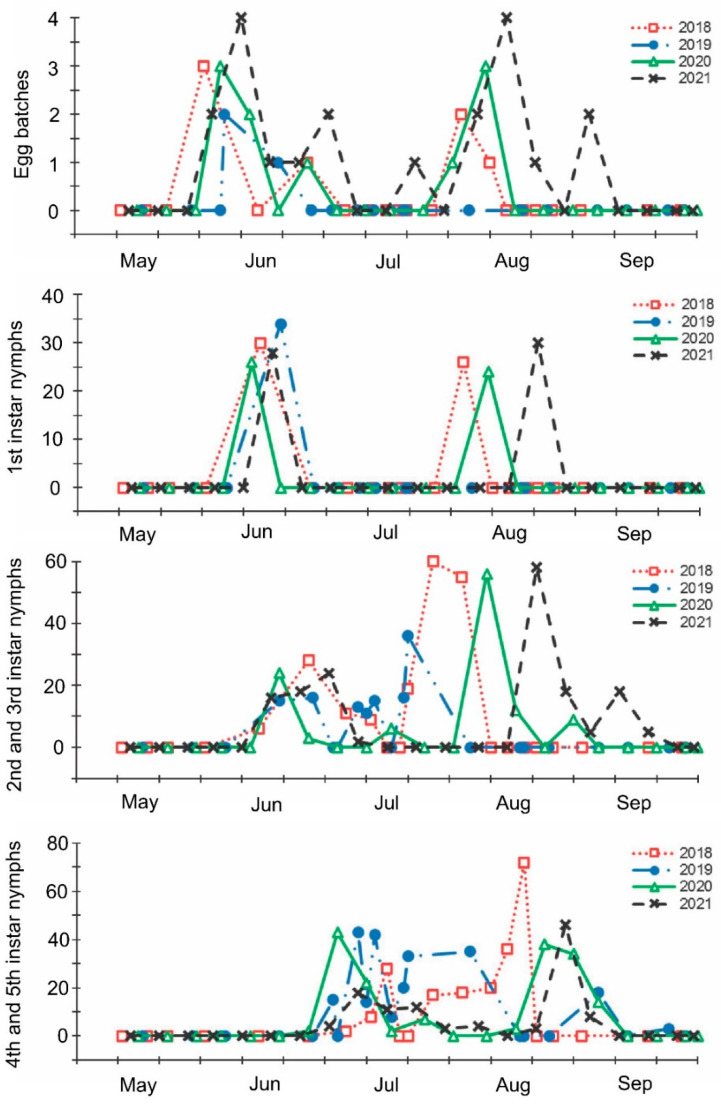
Seasonal changes in numbers of *Halyomorpha halys* egg batches and nymphs of different instars per sample during 2018–2021. Each symbol corresponds to one sample.

The mass oviposition by overwintered females started at the end of May and the beginning of June and lasted until the beginning of the end of July, depending on the year (Figure 2, Supplementary Figure S1). The second peak of mass oviposition was observed in August. In a relatively cool summer of 2021, both periods of egg production were delayed in comparison with the hot summer of 2018 (Figure 2, Supplementary Figure S1). Two peaks in abundance of nymphs of the first instar practically coincided with the two periods of intensive oviposition. Two periods of the highest abundance of nymphs of the second and third instars were observed in June–July (somewhat later than the first periods of mass oviposition and nymph emergence) and, after a short break, again in July–August (Figure 2). Older nymphs were recorded from the end of June to the beginning of September, with a small gap between two peaks at the end of July and the beginning of August (Figure 2). The number of regularly collected adults was almost constant from May to July but gradually increased during August (Supplementary Figure S2).

**Figure 3:**
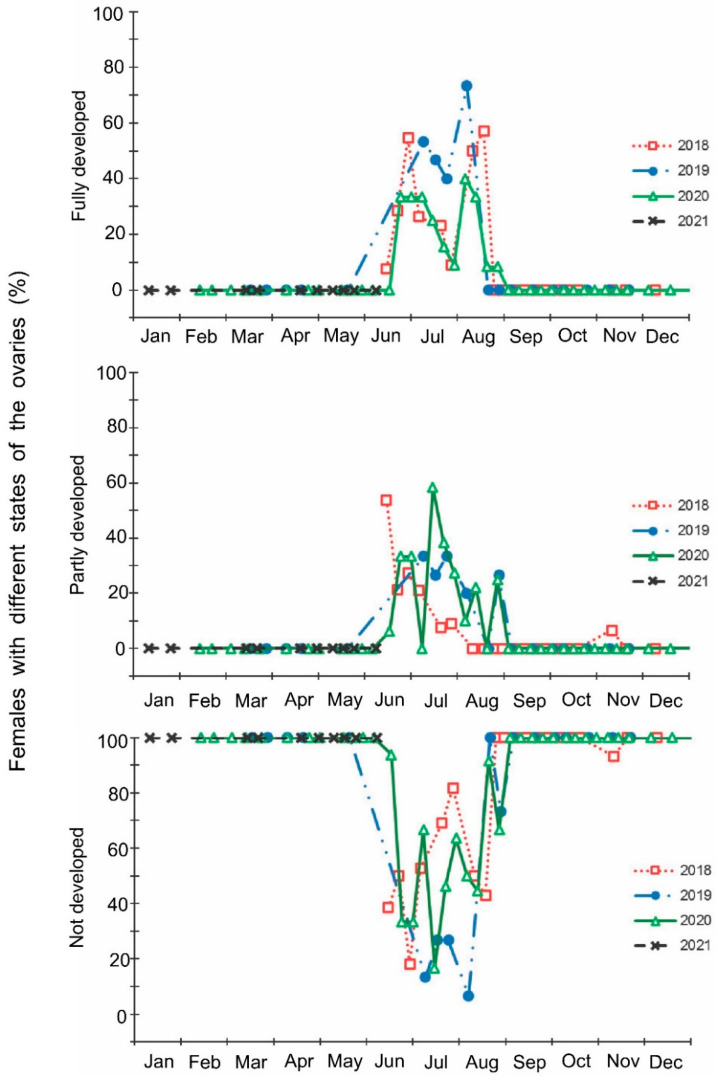
Seasonal changes in the proportions of *Halyomorpha halys* females with different states of the ovaries during 2018–2021. Each symbol corresponds to one sample of field-collected females (6–35 females per sample with a total of 1180 females in 85 samples).

**Figure 4:**
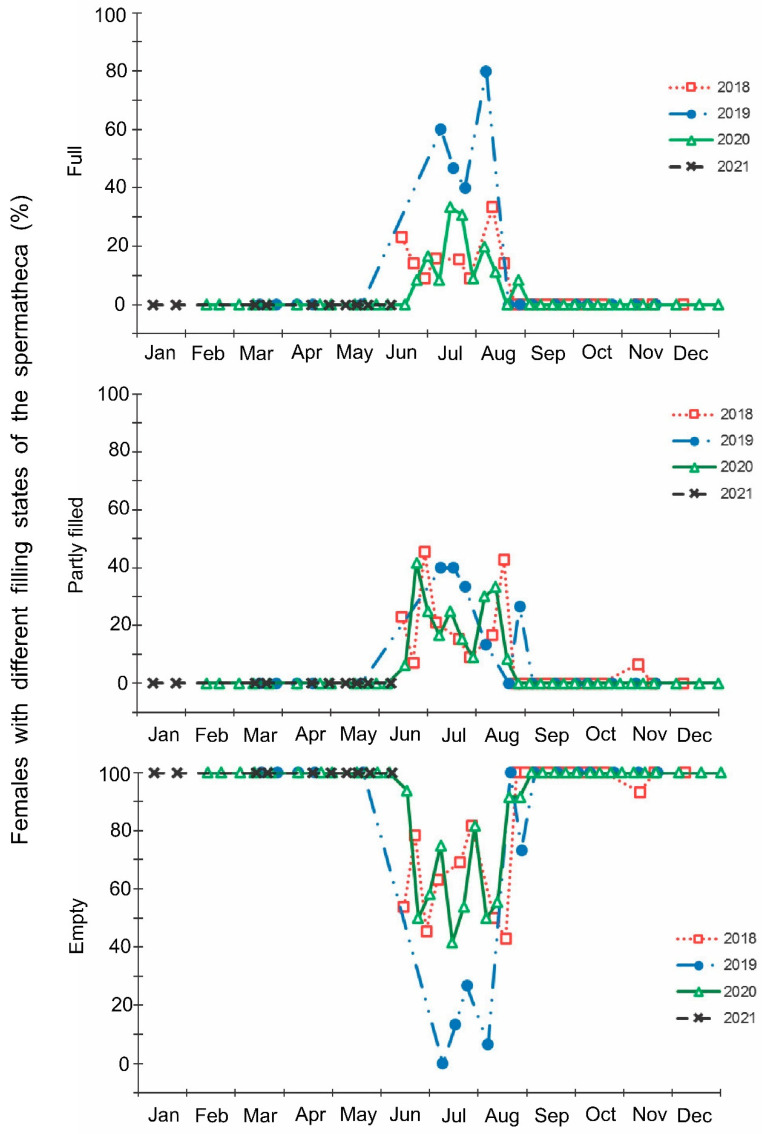
Seasonal changes in the proportions of *Halyomorpha halys* females with different filling states of the spermatheca during 2018–2021. Each symbol corresponds to one sample of field-collected females (6–35 females per sample with a total of 1180 females in 85 samples).

**Figure 5:**
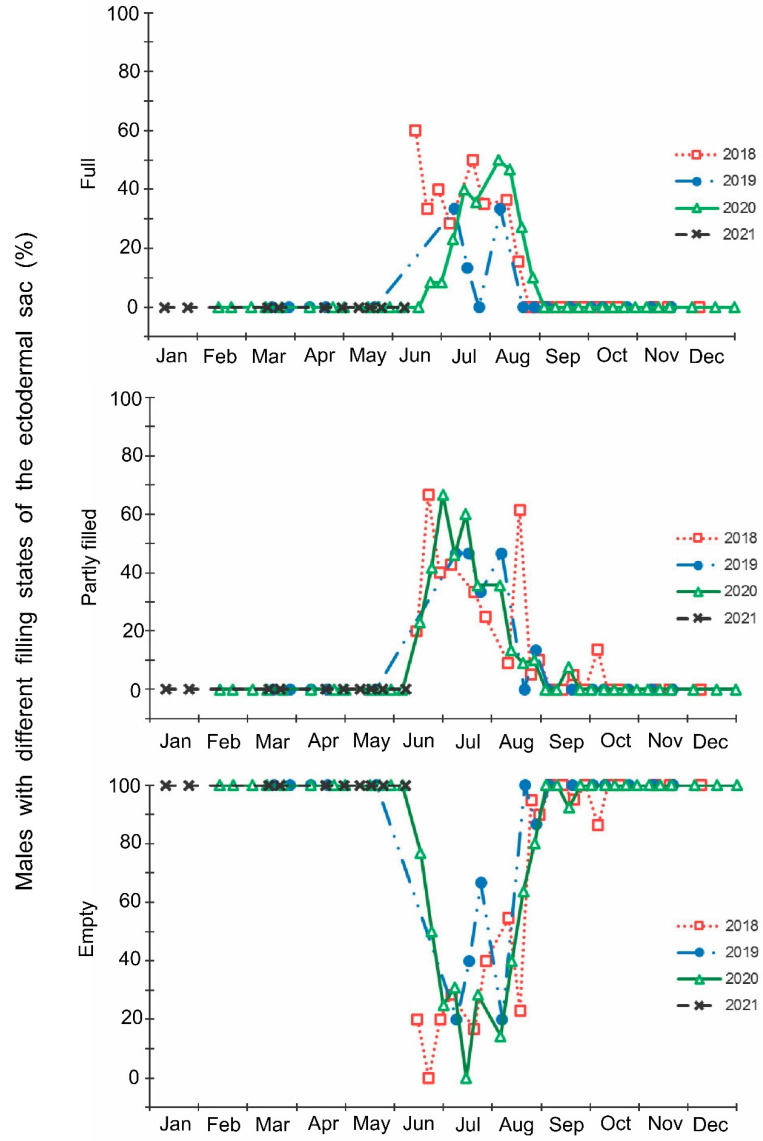
Seasonal changes in the proportions of *Halyomorpha halys* males with different states of the ectodermal sac during 2018–2021. Each symbol corresponds to one sample of field-collected males (3–29 males per sample with a total of 1154 males in 85 samples).

**Figure 6:**
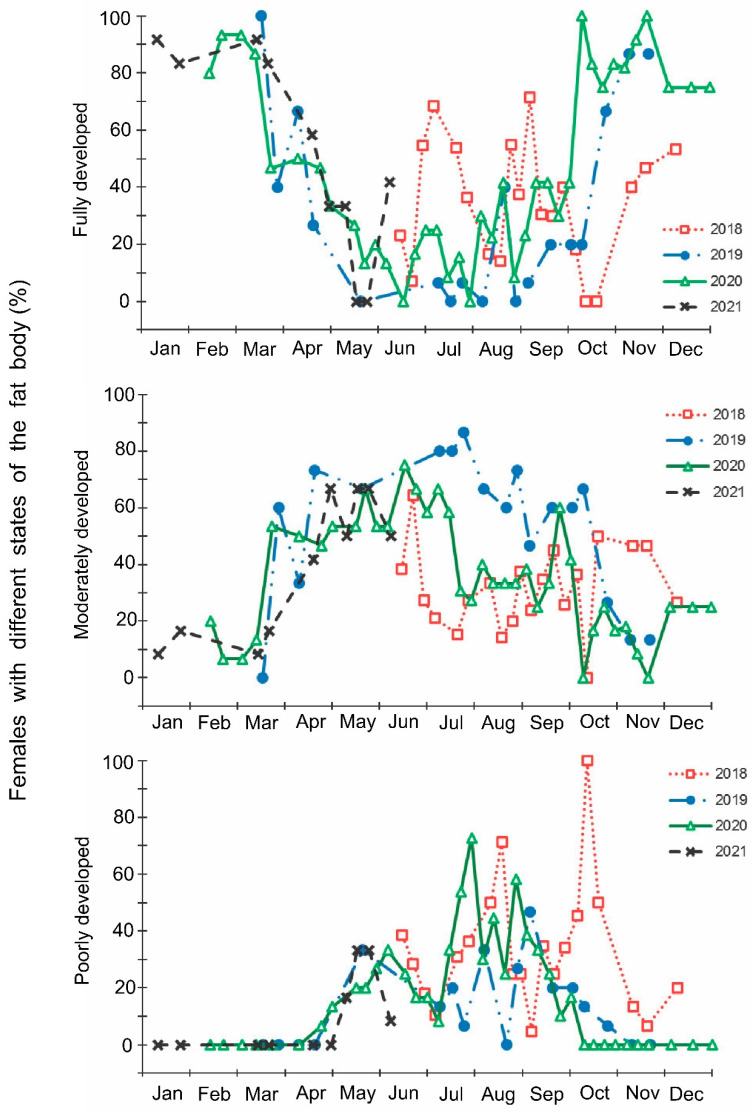
Seasonal changes in the proportions of *Halyomorpha halys* females with different states of fat body during 2018–2021. Each symbol corresponds to one sample of field-collected females (6–35 females per sample with a total of 1180 females in 85 samples).

**Figure 7:**
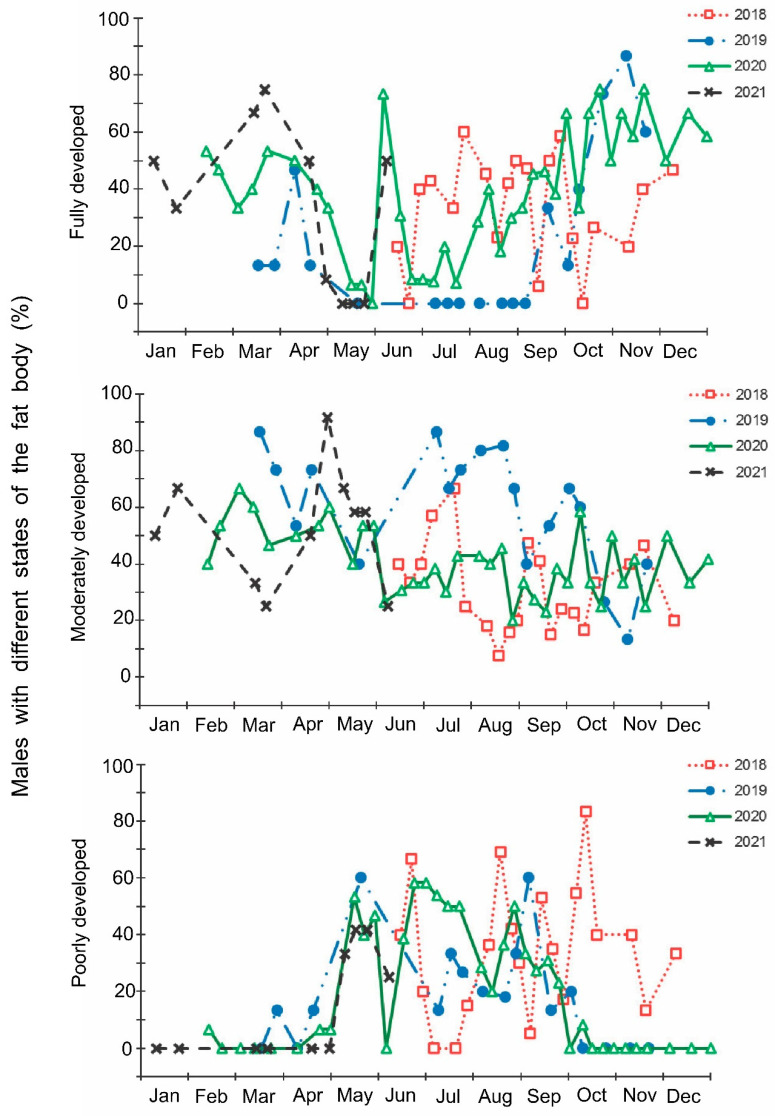
Seasonal changes in the proportions of *Halyomorpha halys* males with different states of fat body during 2018–2021. Each symbol corresponds to one sample of field-collected males (3–29 males per sample with a total of 1154 males in 85 samples).

The laboratory dissections of adults revealed that females with fully or partially developed ovaries and fully or partly filled spermatheca were present from the end of May to the beginning of September. During the rest of the year, almost all dissected females were in a nonreproductive (i.e., diapause) state (Figure 3 and Figure 4). A small drop in the proportions of females with fully developed ovaries and fully filled spermatheca fell on the end of July (Figure 3 and Figure 4). The proportions of males with fully or partly filled ectodermal sacs demonstrated almost the same seasonal pattern, although the decrease in such males in the mid-summer was absent (Figure 5). The seasonal changes in the degree of fat body development in both females and males were mostly opposite to those of their reproductive organs but much less pronounced, although an increase in the proportion of females and males with a fully developed fat body was quite clear in winter (Figure 6 and Figure 7).

**Figure 8:**
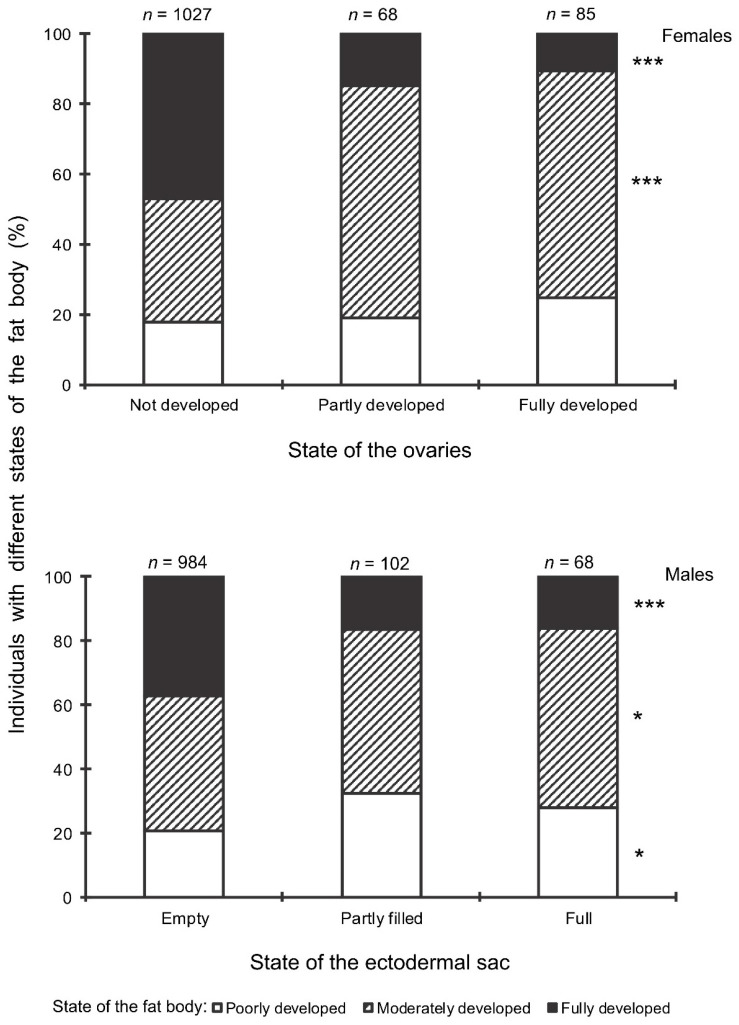
Correlation between the state of reproductive organs and the state of fat body in *Halyomorpha halys* females and males. Spearman’s correlation coefficients for the total data: females: ρ = −0.194 ± 0.023 (*n* = 1180); males: ρ = –0.149 ± 0.027 (*n* = 1154). Sample sizes for the fractions with different states of reproductive organs are indicated above the bars. Asterisks to the right of the graph indicate significant difference in the proportions of individuals with the given state of fat body between males with different states of the ectodermal sack as well as between females with different states of the ovaries (sections with the same fill pattern of different bars of the same graph): *—*p* < 0.05, ***—*p* < 0.001 by the Chi-square test.

The correlation between the state of the reproductive organs and the state of the fat body in *H. halys* females and males was negative: the proportion of individuals with a fully developed fat body among females with undeveloped ovaries and males with empty ectodermal sacs was significantly higher than in reproductively active adults (Figure 8).

**Figure 9:**
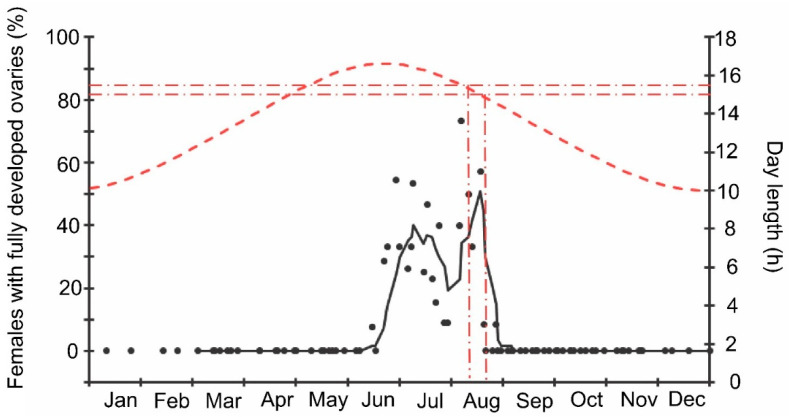
Seasonal changes in the proportions of *Halyomorpha halys* females with fully developed ovaries during 2018–2021 in relation to natural day length. Upper graph, right vertical axis, dashed red line—natural day length including civil twilight [45]. Lower graph, left vertical axis, black symbols—the proportions of *H. halys* females with fully developed ovaries (data of four seasons are pooled, each symbol corresponds to one sample of field-collected females (6–35 females per sample with a total of 1180 females in 85 samples)), black trend line is a moving average of five points (produced in Excel 2016). Red dash-dotted lines show the threshold zone of *H. halys* photoperiodic response of winter adult diapause induction estimated from the earlier laboratory experiments (15.0–15.5 h [16]) and its projection to the calendar axis.

Finally, it should be noted that the above-mentioned seasonal changes were evidently correlated with day length: when the natural photoperiod decreased below the experimentally determined critical day length (15–15.5 h [16]), the proportion of females with fully developed ovaries sharply decreased to zero (Figure 9).

We further compared the data from the field sampling in the Sochi region with the results of the early laboratory experiments involving individuals from *H. halys* from the same local population. The experiments demonstrated that the sum of effective temperatures (SET) required for pre-adult development under the long-day conditions was about 590 degree-days, calculated above the lower development threshold of 13.3 °C [16]. The thermal requirements for female maturation (=time from the emergence of a female to first oviposition) were not determined in the above-cited study, and therefore we use the parameter (156 degree-days) calculated based on the data reported elsewhere [55]. In total, the full life cycle yielded about 746 degree-days.

## 4. Discussion

As noted above, populations of the brown marmorated stink bug are bivoltine or partially bivoltine throughout most of the species’ native [12,17,18,30] and invasive [11,23,28,29,33,34,35,36,37] geographic ranges, although in some colder regions only one generation per year is produced [19,25,30,56,57]. The results of the regular field sampling during the four consecutive years and the monitoring of several morphological, physiological, and demographic parameters clearly indicate that in the Sochi region, *H. halys* is at least partially bivoltine: the main period of mass oviposition (by the overwintered females) occurs in June–July; the second much shorter period of egg-laying (by females of the new, i.e., the first, generation) followed by a new peak in the abundance of early instar nymphs occurs in August (Figure 2). Considering that under the long-day conditions and temperatures of 20–25 °C, the maturation (i.e., pre-oviposition period) of *H. halys* females lasts approximately 15–20 days [16,47,56,57], we can conclude that the mass emergence of a new (i.e., the first) generation of adults occurs in July. Thus, the prediction made earlier based on the data from sporadic field observations [15] and the results of the laboratory experiments [16] were confirmed in this study.

Somewhat more advanced phenology has been reported for native Chinese bivoltine populations: the overwintered females begin ovipositing in mid or late May, whereas the first-generation adults start to lay eggs from June to July [30]. In North American invasive populations, oviposition by the overwintered females starts, depending on the climate zone, from May to June, and adults of the first generation emerge from June to July [35], which is also somewhat earlier than observed for the Sochi population.

The beginning of egg-laying by the overwintered females of the invasive Sochi population is evidently determined by temperature: in warmer springs, oviposition starts earlier than in colder springs (Figure 1 and Figure 2; Supplementary Figure S1), and in all cases, it happens almost two months after the moment when the natural day length becomes longer than the critical photoperiod (Figure 9). The same is evident in the comparison of data from different climatic zones: in the warmer regions within the native range of *H. halys*, its oviposition starts in May [30], whereas, in the colder climate, females of the invasive population lay their first eggs only in June [19]. The termination of oviposition, however, is controlled by the photoperiod, as strongly supported by the close coincidence with the moment when the natural day length decreases to the experimentally determined critical value (15.0–15.5 h [16]). It should be noted that the number of the late- and even early-instar nymphs collected during the field sampling was rather high until September (Figure 2), indicating that a significant fraction of females enter winter diapause without laying eggs in the current season and, moreover, some nymphs possibly fail to reach the adult stage and prepare for overwintering in time, and they are likely destined to die during the winter. This seasonal pattern has been observed in several other Pentatomidae species [4].

In this study, several morphological, physiological, and demographic parameters were simultaneously monitored and analyzed (Figure 2, Figure 3, Figure 4, Figure 5, Figure 6 and Figure 7, Supplementary Figure S2). Most clearly, the phenology and timing of reproductive events are reflected by the degree of development in the ovarioles of females (Figure 3), ectodermal sacs in males (Figure 5), and abundance of early nymphal instars (Figure 2). The dynamics of the state of the fat body in both sexes seem to be less informative (Figure 6 and Figure 7), although the correlation between the state of reproductive organs and the state of the fat body is statistically significant (Figure 8). This observation might indicate that when food is easily available to this highly polyphagous pentatomid, even reproductive (i.e., nondiapause) adults might form a fat body.

Finally, we compared the data from the field sampling in the Sochi region with the results of the early laboratory experiments involving the same local population of *H. halys*. The full life cycle (from the oviposition of the parental generation to the first egg mass produced) yielded about 746 degree-days. The first peak of oviposition (by the overwintered females) under natural conditions in the Sochi region was observed at the beginning of June, and the second peak (supposedly by females of the new generation) was observed in the middle of August (Figure 2). Calculations based on the mean daily temperatures recorded in the studied region show that the SET accumulated during this period varied, depending on the year, from 730 to 790 degree-days (Supplementary Figure S1) which fits well with regard to the above calculations. Moreover, the SET accumulated from the middle of August till the end of October (when the mean daily temperatures drop below the lower development threshold of *H. halys*) varied from 540 to 670 degree-days which, according to the above experimental results, is sufficient for pre-adult development but not for female maturation. These calculations suggest that most (or at least a substantial fraction of) adults of the second generation have enough time to enter diapause and thereby prepare for overwintering, but practically none of the second-generation females a have chance to lay eggs before winter.

Summarizing the fundamental aspect of the study, we conclude that both the rate of *H. halys* pre-adult development and the timing of the induction of winter adult diapause observed under the natural conditions agree with the predictions based on the results of the laboratory experiments conducted earlier. Similar coherence of results was obtained, for example, for the blue blowfly, *Calliphora vicina* R.-D. (Diptera: Calliphoridae) [58], a tortricid moth *Adoxophyes orana* (Fischer von Röslerstamm) (Lepidoptera: Tortricidae) [59], wheat stink bugs, *Aelia sibirica* Reuter and *A. acuminata* (L.) [60], red-banded shield bug, *Piezodorus hybneri* (Gmelin) [61], and other insect species studied in sufficient detail both in the field and laboratory [1,2,4].

In this study, we utilized a widely used simple method of SET calculation based on the data of daily mean temperature. More precise calculations such as the single or double sine method or more sophisticated models [62,63] can probably improve the analysis (in particular, if the raw data presented in Tables S1–S8 are used). These results might be useful for the development of regional strategies to monitor and control this important agricultural pest as well as predicting its further spread in Europe and elsewhere, as a part of both the current trans-continental invasion and in response to the ongoing climate change [64].

## Data Availability

Data supporting reported results can be obtained upon request to the corresponding author (D.L.M.).

## Supplementary Materials

The following supporting information can be downloaded at: https://www.mdpi.com/article/10.3390/insects13070580/s1, Figure S1: Seasonal dynamics of the accumulation of the sum of effective temperatures (SET) with the lower developmental threshold of 13.3 °C, seasonal changes in numbers of *Halyomorpha halys* egg batches and the first instar nymphs per sample during 2018–2021 plotted against the accumulated SET; Figure S2: Seasonal changes in numbers of *Halyomorpha halys* adults per sample during 2018–2021; Table S1: Min and max temperatures for 2018–2021 in Sochi, Russia (raw data for Figure 1); Table S2: Seasonal changes in numbers of *Halyomorpha halys* egg batches, nymphs, and of different instars per sample during 2018–2021 (raw data for Figure 2 and Figure S1); Table S3: Seasonal changes in the proportions of *Halyomorpha halys* females with different states of the ovaries during 2018–2021 (raw data for Figure 3); Table S4: Seasonal changes in the proportions of *Halyomorpha halys* females with different filling states of the spermatheca during 2018–2021 (raw data for Figure 4); Table S5: Seasonal changes in the proportions of *Halyomorpha halys* males with different states of the ectodermal sac during 2018–2021. Each symbol corresponds to one sample of field-collected males (raw data for Figure 5); Table S6: Seasonal changes in the proportions of *Halyomorpha halys* females with different states of fat body during 2018–2021 (raw data for Figure 6); Table S7: Seasonal changes in the proportions of *Halyomorpha halys* males with different states of fat body during 2018–2021 (raw data for Figure 7); Table S8: Seasonal dynamics of the accumulation of the sum of effective temperatures (SET) with the lower developmental threshold of 13.3 °C, seasonal changes in numbers of *Halyomorpha halys* egg batches and the first instar nymphs per sample during 2018–2021 plotted against the accumulated SET (raw data for Figure S2).
