# Supplementary material for: To Every Thing There Is a Season: Phenology and Photoperiodic Control of Seasonal Development in the Invasive Caucasian Population of the Brown Marmorated Stink Bug, Halyomorpha halys (Hemiptera: Heteroptera: Pentatomidae)
Source: Insects. 2022 Jun 25;13(7):580. doi: 10.3390/insects13070580 (PMC9323183; doi:10.3390/insects13070580)
Supplement: Supplementary file 1 [file insects-13-00580-s001.zip › H_halys-phenology-Suppl_Figure_S1.pdf]

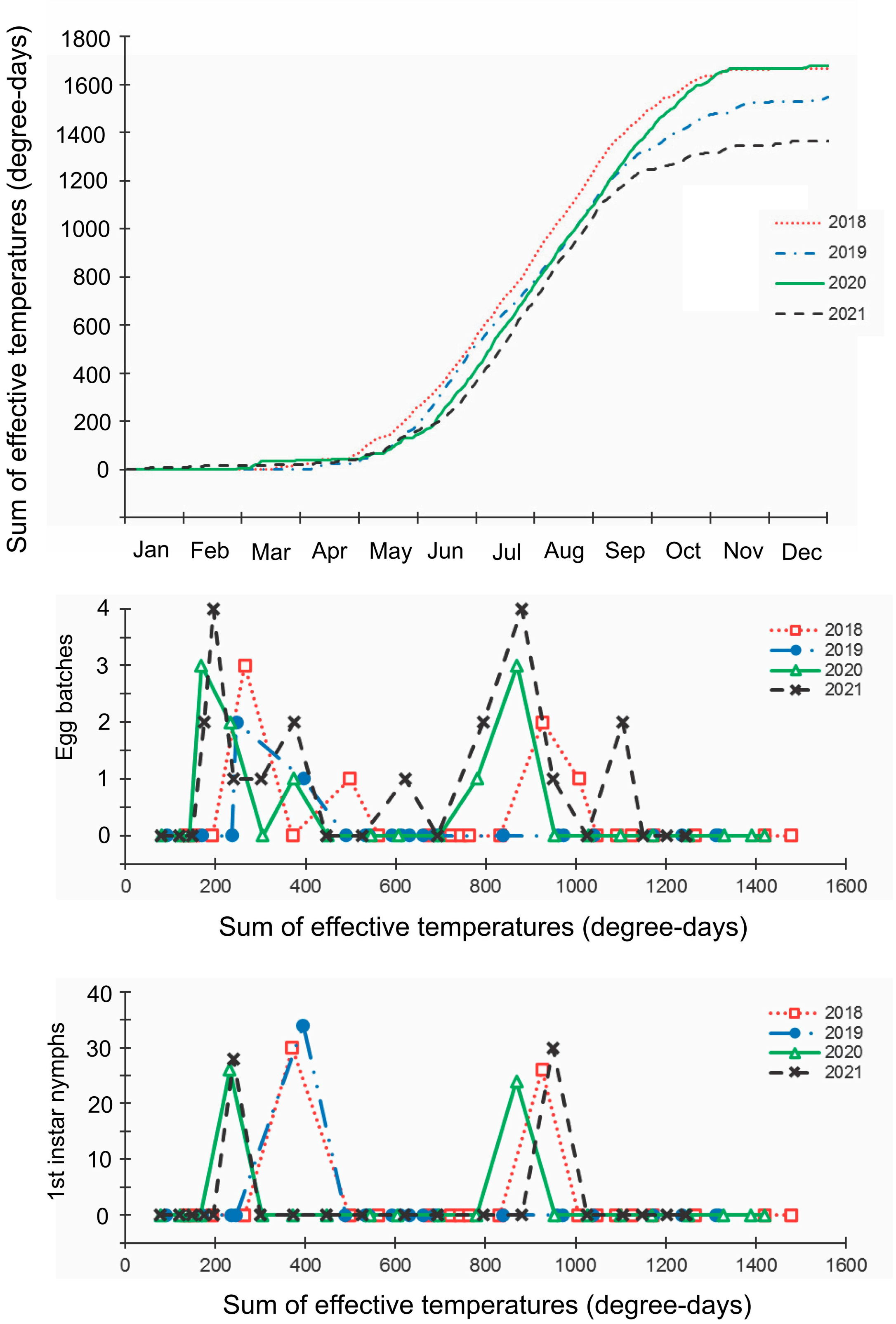

Figure S1. Top graph, **s**easonal dynamics of the accumulation of the sum of effective temperatures (SET) with the lower developmental threshold of 13.3°C; middle and bottom graphs, seasonal changes in numbers of *Halyomorpha halys* egg batches and the first instar nymphs per sample during 2018–2021 plotted against the accumulated SET. Each symbol corresponds to one sample.
