# Supplementary material for: To Every Thing There Is a Season: Phenology and Photoperiodic Control of Seasonal Development in the Invasive Caucasian Population of the Brown Marmorated Stink Bug, Halyomorpha halys (Hemiptera: Heteroptera: Pentatomidae)
Source: Insects. 2022 Jun 25;13(7):580. doi: 10.3390/insects13070580 (PMC9323183; doi:10.3390/insects13070580)
Supplement: Supplementary file 1 [file insects-13-00580-s001.zip › H_halys-phenology-Suppl_Figure_S2.pdf]

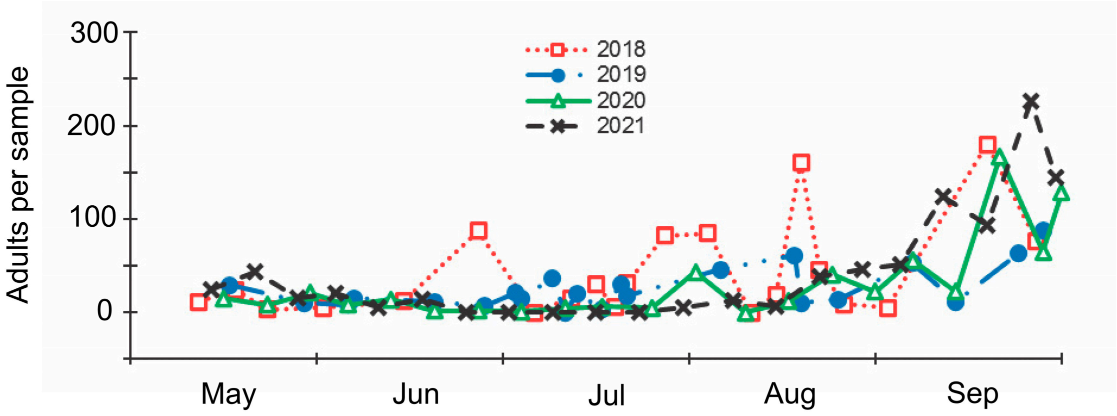

Figure S2. Seasonal changes in numbers of *Halyomorpha halys* adults per sample during 2018–2021. Each symbol corresponds to one sample.
